# The Guardianship of Children

**Published:** 1921-05-14

**Authors:** 


### The Guardianship of Children.

Since the Government gave general support to the Guardianship of Infants Bill, which was read a second time last week in the House of Commons, there is good hope that this measure will soon become law, though it is likely to undergo some modification in Committee. The Bill deals with that branch of the law relating to family and domestic relations in regard to parent and child. It is aimed at the grievance that under the existing law, when any difference or dispute occurs between father and mother as to the guardianship, maintenance, and education of their children, assuming that there are no serious charges of misconduct against the father, the father's wishes must prevail. Thus the scales of justice are, in a sense, unduly weighted in favour of the father, and it is an occasional experience that the health and well-being of the children suffer greatly through the arbitrary operation of the existing law.

The whole tendency of recent legislation—as Colonel Grey, who moved the second reading, reminded the House—has been to put man and woman, husband and wife, on an equality, both as to their property and their other civil rights, and this Bill embodies the claim that they should be placed on an equal footing in the control and upbringing of their children. It also proposes to place on the Statute-book a clear and definite statutory obligation on parents to maintain and bring up their children according to their respective means. In the Press a good deal has been made of the difficulty that might arise in cases where there is only one infant, thus compelling the Court to resort to the example of Solomon. But the answer to that is that in practice no such difficulty has arisen under the administration of a similar statute in the Colonies. Lady Astor, who, on behalf of women's organisations, supported the Bill whole-heartedly and with characteristic wit and force, put the matter in a nutshell when she pictured the plight of a woman who has unfortunately married a "rotter," and who realises that she has to keep a large family which is under the entire guardianship of a man who has shown himself to be a bad father and a bad husband.

There is, of course, the danger that a measure of this kind will increase the wrangling between husbands and wives in the Courts. On the other hand, it may keep many cases out, because under its provisions the husband would know that the wife would get equal consideration. One of its most useful clauses is that for improving the machinery by which maintenance orders are enforced, so that it becomes considerably more difficult for a man to avoid his legal obligations to his wife and children. On the whole, the Bill is, we think, definitely in the interests of the children.

